# First Report of *RET* Fusion‐Positive Lung Adenocarcinoma With Miliary Intrapulmonary Metastases Responsive to Selpercatinib: A Case Report

**DOI:** 10.1002/ccr3.71011

**Published:** 2025-10-04

**Authors:** Soichi Iwanaka, Tadashi Nishimura, Hajime Fujimoto, Daichi Morita, Hitoshi Kuru, Yasumasa Sakakura, Corina N. D'Alessandro‐Gabazza, Taro Yasuma, Esteban C. Gabazza, Masahiro Naito, Hidenori Ibata, Tetsu Kobayashi

**Affiliations:** ^1^ Department of Pulmonary Medicine Mie Chuo Medical Center Tsu Mie Japan; ^2^ Department of Pulmonary and Critical Care Medicine Mie University Faculty and Graduate School of Medicine Tsu Mie Japan; ^3^ Department of Immunology Mie University Faculty and Graduate School of Medicine Tsu Mie Japan

**Keywords:** miliary lung metastases, non‐small cell lung cancer, *RET* fusion, selpercatinib

## Abstract

Lung cancer presenting with miliary lung metastases on chest computed tomography is associated with poor prognosis. Recent studies indicate that non‐small cell lung cancer harboring driver gene mutations, including rearranged during transfection (*RET)* fusions, can manifest with miliary lung metastases. However, the efficacy of molecularly targeted therapies in *RET* fusion‐positive non‐small cell lung cancer with miliary dissemination remains unknown. Here, we report a case of a 45‐year‐old man with a right lower lobe lung nodule, bilateral miliary intrapulmonary metastases, extensive lymphadenopathy, and metastases to the left adrenal gland, liver, and multiple bones. Transbronchial needle aspiration confirmed lung adenocarcinoma, with *RET* fusion detected via gene panel testing. Treatment with selpercatinib resulted in a sustained partial response for over one year, providing the first clinical evidence supporting this targeted therapy in *RET* fusion‐positive lung adenocarcinoma with miliary metastases.

**Summary**

- Targeted therapy with selpercatinib can achieve disease control even in cases with miliary intrapulmonary metastases, offering a promising treatment option for *RET* fusion‐positive NSCLC.
- This case underscores the critical role of molecular profiling in guiding personalized therapy, optimizing patient outcomes, and expanding treatment possibilities for advanced lung cancer.

## 1 Introduction

Miliary shadows on computed tomography (CT) are characterized by the presence of diffuse, small, discrete pulmonary nodules measuring 1–2 mm in diameter [1, 2]. This radiological pattern is most commonly associated with infectious, occupational, and inflammatory diseases, including miliary tuberculosis, pneumoconiosis, sarcoidosis, and hypersensitivity pneumonitis, and metastatic dissemination from hypervascularized tumors [1, 3]. Notably, malignant lung tumors, including non‐small cell lung cancer (NSCLC), rarely exhibit this pattern at initial presentation.

Emerging evidence suggests that intrapulmonary metastases presenting with miliary shadows are frequently linked to an unfavorable prognosis [4]. Interestingly, recent studies have reported a higher prevalence of miliary lung dissemination in NSCLC cases harboring specific driver gene mutations, such as epidermal growth factor receptor (EGFR) mutations and the rare rearranged during transfection (*RET*) fusion [5, 6]. However, despite these observations, no studies to date have evaluated whether the clinical outcome of NSCLC with miliary dissemination driven by *RET* fusion can be improved through molecularly targeted therapies. Addressing this gap is critical for optimizing therapeutic strategies and improving clinical outcomes in this unique subset of patients.

Here, we report a case of NSCLC harboring a *RET* fusion gene that presented with a miliary pattern on chest CT. The patient was treated with a RET‐targeted tyrosine kinase inhibitor, demonstrating a marked radiological and clinical response. This case highlights the potential efficacy of molecularly targeted therapies in NSCLC with RET fusion and extensive intrapulmonary dissemination, underscoring the need for further investigation into personalized treatment strategies for this distinct clinical phenotype.

## 2 Case History and Examination

The patient was a 45‐year‐old male with a 17‐pack‐year smoking history who presented to our department with a 2‐month history of progressively worsening persistent cough accompanied by right supraclavicular lymphadenopathy. On physical examination, his vital signs were within normal physiological ranges, including stable blood pressure, heart rate, respiratory rate, and body temperature. Palpation of the cervical region revealed no evidence of lymphadenopathy or abnormal masses in the cervical triangles. Pulmonary examination demonstrated clear lung sounds on auscultation.

**FIGURE 1:**
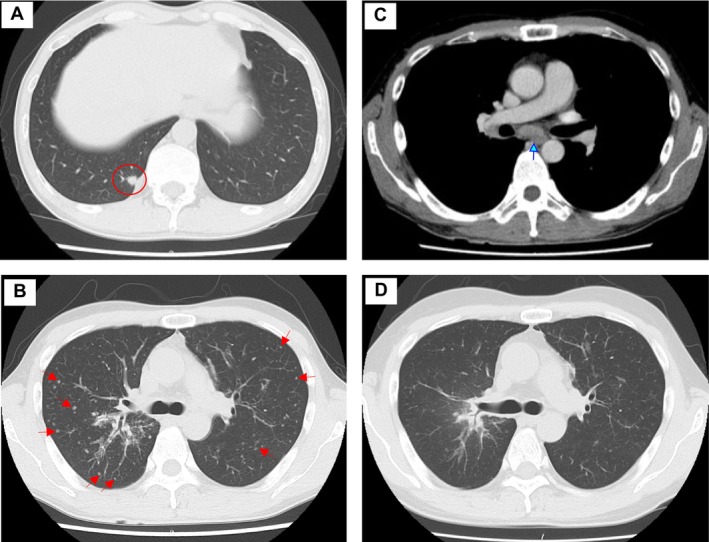
Chest computed tomography (CT) findings. CT revealed nodules in the right lower lobe (A) and miliary shadows in both lungs (B). The red circle denotes the primary lung tumor; red arrows indicate minimal intrapulmonary metastases, and the blue arrow highlights enlarged subcarinal lymph nodes. Contrast‐enhanced chest CT demonstrated enlarged subcarinal lymph nodes (C). After 11 months, the tumor showed a partial but stable response (D).

Chest CT revealed nodular lesions in the right lower lung lobe, diffuse miliary opacities in both lungs, and multiple enlarged lymph nodes, including subcarinal lymphadenopathy (Figure 1A–C).

## 3 Differential Diagnosis, Investigations, and Treatment

The micronodules observed on CT were independent of the components of the secondary lobule and exhibited a symmetrical, diffuse distribution. Based on these findings, miliary tuberculosis and highly vascular tumors, such as renal cell carcinoma, were considered. Alternative differential diagnoses included sarcoidosis and metastatic lung tumors. Blood tests revealed elevated carcinoembryonic antigen (10.7 ng/mL) and sialyl Lewis X (470 U/mL), while angiotensin‐converting enzyme and soluble interleukin‐2 receptor levels were within normal ranges. The interferon‐gamma release assay was negative. Contrast‐enhanced CT further demonstrated metastatic involvement of the left adrenal gland, liver, and multiple bones.

**FIGURE 2:**
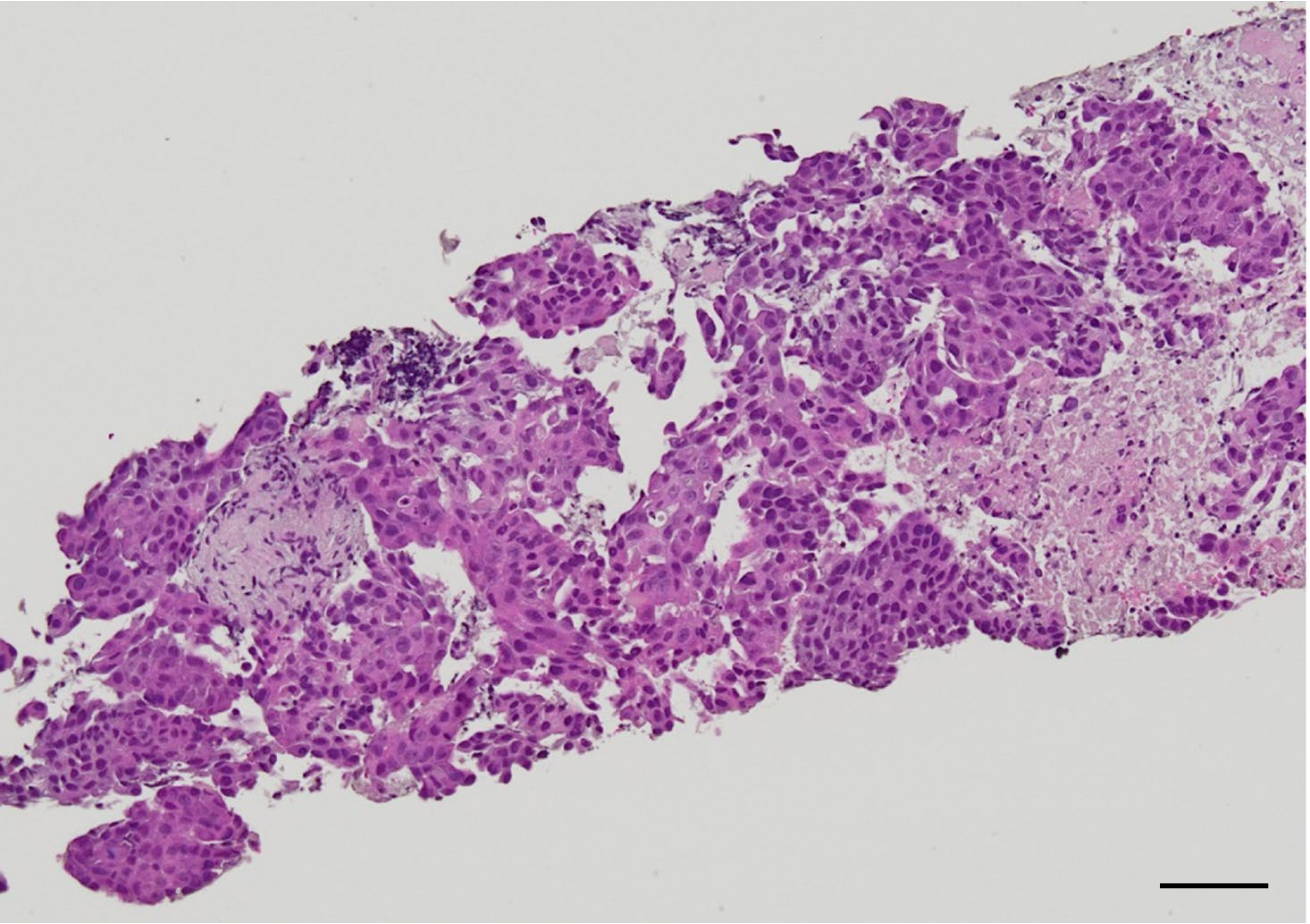
Histopathological findings. Hematoxylin and eosin staining revealed characteristic features of lung adenocarcinoma.

To establish a definitive diagnosis, endobronchial ultrasound‐guided transbronchial needle aspiration was performed on a subcarinal lymph node. Histopathological examination confirmed lung adenocarcinoma, and multiplex gene panel testing identified a RET fusion (Figure 2). Following diagnosis, the patient was initiated on treatment with the selective RET inhibitor selpercatinib.

## 4 Conclusions and Results

Follow‐up imaging demonstrated a partial response to treatment (Figure 1D), with the disease remaining stable for over a year. The patient has exhibited sustained clinical improvement with no significant disease progression or treatment‐related adverse events. He maintains a good performance status and is preparing to return to work, indicating a favorable therapeutic response to selpercatinib. Regular follow‐up assessments are ongoing to monitor long‐term disease control and treatment efficacy.

## 5 Discussion

To our knowledge, this is the first published case to explicitly describe a miliary pattern in RET fusion‐positive NSCLC responding to selpercatinib. Lung cancer typically manifests on imaging as nodular lesions, pleural effusions, and lymphadenopathy [7]. However, miliary shadows are an uncommon radiological presentation, occurring in approximately 2% of NSCLC cases at initial diagnosis [4]. The pathophysiological mechanism underlying this pattern involves initial hematogenous dissemination, potentially originating from primary tumors or metastatic sites, leading to the release of tumor emboli into the pulmonary circulation [4]. While bone metastases have been suggested as a potential intermediate step in this process, reports indicate that miliary lung metastases can also occur independently of bone involvement, possibly through direct hematogenous spread from other extrapulmonary metastatic sites [4, 8].

Several studies have reported an association between miliary intrapulmonary metastases and NSCLC harboring driver gene alterations, including EGFR mutations, ALK and RET fusions, and ROS1 rearrangements [6, 9, 10, 11]. In tumors with EGFR mutations, paracrine signaling loops between cancer cells and surrounding stromal cells have been implicated in promoting widespread metastatic spread [12]. Similarly, in medullary thyroid carcinoma, RET mutations are associated with elevated expression of the extracellular matrix protein tenascin‐C, which contributes to tumor invasiveness and poorer survival outcomes [13]. Despite these insights, the mechanisms by which genetic alterations drive metastatic dissemination remain incompletely understood. The prognosis of NSCLC presenting with miliary dissemination has generally been considered poor. However, a recent study investigating the efficacy of EGFR‐tyrosine kinase inhibitors (TKIs) in this subgroup reported no statistically significant difference in overall survival between patients with or without miliary metastases, suggesting that molecularly targeted therapies may improve clinical outcomes in these patients [9].

*RET* gene rearrangements are identified in approximately 1%–2% of NSCLC cases [14]. The clinical efficacy and safety of selpercatinib, a highly selective RET kinase inhibitor, were first established in the LIBRETTO‐001 trial, a global, single‐arm, phase 1–2 study that enrolled patients with RET fusion‐positive NSCLC and medullary thyroid cancer [15, 16]. In this trial, selpercatinib demonstrated a high objective response rate of 84% and a median progression‐free survival of 22.0 months, underscoring its therapeutic potential in RET‐driven NSCLC [17]. Building on these findings, the phase III LIBRETTO‐431 trial provided robust confirmatory evidence, showing that selpercatinib significantly prolonged PFS compared to platinum‐based chemotherapy, with or without immune checkpoint inhibitors, in patients with advanced RET fusion‐positive NSCLC [18]. Specifically, selpercatinib achieved a median progression‐free survival of 24.8 months, compared to 11.2 months in the control group (hazard ratio, 0.48; 95% CI, 0.33–0.70; *p* < 0.001) [18]. Complementing the results from clinical trials, the retrospective SIREN study reported an objective response rate of 68% and a median progression‐free survival of 15.6 months, further substantiating the clinical benefit of selpercatinib in real‐world settings [19]. Additionally, Sarfaty et al. documented a case series involving 14 patients with RET fusion‐positive NSCLC, 8 of whom exhibited miliary pulmonary nodules, radiological findings reminiscent of our present case [5]. Notably, none of the cases in that series were treated with selpercatinib, and thus its specific impact on miliary‐pattern metastasis remains to be elucidated [5].

In the present case, our patient exhibited a durable response to selpercatinib, maintaining disease control for over 1 year despite extensive bilateral miliary lung metastases. This case provides novel evidence suggesting that *RET*‐targeted therapy can significantly improve clinical outcomes even in patients with diffuse intrapulmonary dissemination, underscoring the need for further investigation into optimized treatment strategies for this distinct NSCLC phenotype.

## 6 Conclusion

This case highlights the critical importance of comprehensive driver gene testing, including *RET* fusion analysis, in NSCLC patients presenting with miliary lung metastases and systemic disease dissemination. Our findings underscore that even in cases with extensive intrapulmonary and extrapulmonary metastases, targeted therapy with selpercatinib can offer a promising therapeutic option, potentially transforming prognosis and clinical outcomes in *RET* fusion‐positive NSCLC. These results underscore the importance of molecular profiling in guiding precision oncology strategies, ensuring that patients with aggressive disease phenotypes receive the most effective, individualized treatment approaches.

## Author Contributions

**Soichi Iwanaka:** writing – original draft. **Tadashi Nishimura:** writing – original draft. **Hajime Fujimoto:** writing – original draft. **Daichi Morita:** writing – original draft. **Hitoshi Kuru:** writing – original draft. **Yasumasa Sakakura:** writing – original draft. **Corina N. D'Alessandro‐Gabazza:** writing – original draft. **Taro Yasuma:** writing – original draft. **Esteban C. Gabazza:** writing – original draft. **Masahiro Naito:** writing – original draft. **Hidenori Ibata:** writing – original draft. **Tetsu Kobayashi:** writing – original draft.

## Ethics Statement

The Mie Chuo Medical Center Review Board approved the submission of this case report (approval No: CR2024‐05).

## Consent

Written informed consent was obtained from the patient for the publication of this report.

## Conflicts of Interest

The authors declare no conflicts of interest.

## Data Availability

All data from this case report are available upon reasonable request to the first author.
